# Prognostic Value of Four Preimplantation Malnutrition Estimation Tools in Predicting Heart Failure Hospitalization of the Older Diabetic Patients with Right Ventricular Pacing

**DOI:** 10.1007/s12603-023-2042-6

**Published:** 2023-11-30

**Authors:** B. Fu, Y. Yu, S. Cheng, H. Huang, T. Long, J. Yang, M. Gu, C. Cai, X. Chen, H. Niu, Wei Hua

**Affiliations:** 1 Cardiac Arrhythmia Center, National Center for Cardiovascular Diseases, State Key Laboratory of Cardiovascular Disease, Fuwai Hospital, Chinese Academy of Medical Sciences and Peking Union Medical College, No. 167 Bei Li Shi Rd, Xicheng District, 100037, Beijing, China

**Keywords:** Nutritional index, older, diabetes, right ventricular pacing

## Abstract

**Objectives:**

The prognostic value of preimplantation nutritional status is not yet known for older diabetic patients that received right ventricular pacing (RVP). The study aimed to investigate the clinical value of the four malnutrition screening tools for the prediction of heart failure hospitalization (HFH) in older diabetic patients that received RVP.

**Design:**

Retrospective observational cohort study.

**Setting and Participants:**

This study was conducted between January 2017 and January 2018 at the Fuwai Hospital, Beijing, China, and included older (age ≥ 65 years) diabetic patients that received RVP for the first time

**Measurements:**

The Prognostic Nutritional Index (PNI), Geriatric Nutritional Risk Index (GNRI), Naples Prognostic Score (NPS), and the Controlling Nutritional Status (CONUT) score were used to estimate the preimplantation nutritional status of the patients. Univariate and multivariate Cox proportional hazard regression analyses were performed to investigate the association between preimplantation malnutrition and HFH.

**Results:**

Overall, 231 older diabetic patients receiving RVP were included. The median follow-up period after RVP was 53 months. HFH was reported for 19.9% of the included patients. Our results showed preimplantation malnutrition for 18.2%, 15.2%, 86.6% and 66.2% of the included patients based on the PNI, GNRI, NPS, and CONUT score, respectively. The cumulative rate of HFH during follow-up period was significantly higher for patients in the preimplantation malnutrition group based on the PNI (log-rank = 13.0, P = 0.001), GNRI (log-rank = 8.5, P = 0.01), and NPS (log-rank = 15.7, P < 0.001) compared to the normal nutrition group, but was not statistically significant for those in the preimplantation malnutrition group based on the CONUT score (log-rank = 2.7, P = 0.3). As continuous variables, all the nutritional indices showed significant correlation with HFH (all P < 0.05). However, multivariate analysis showed that only GNRI was independently associated with HFH (HR = 0.97, 95% CI: 0.937–0.997, P = 0.032). As categorical variables, PNI, GNRI, and NPS showed significant correlation with HFH. After adjustment of confounding factors, moderate-to-severe degree of malnutrition was an independent predictor of HFH based on the PNI (HR = 4.66, 95% CI: 1.03-21.00, P = 0.045) and GNRI (HR = 3.02, 95% CI: 1.02-9.00, P = 0.047).

**Conclusion:**

Preimplantation malnutrition was highly prevalent in older diabetic patients that received RVP. The malnutrition prediction tools, PNI and GNRI, showed significant prognostic value in accurately predicting HFH in older diabetic patients with RVP.

## Introduction

**T**he number of patients receiving permanent pacemaker implantation (PPMI) has grown significantly because of the constantly increasing global aging population. According to current estimates, approximately 1 million pacemaker devices are implanted every year (1). Right ventricular pacing (RVP) is a conventional PPMI strategy that involves single-site pacemaker implantation in the right ventricular septum (RVS) or the right ventricular apex (RVA) (2). However, RVP is associated with deterioration of left ventricular function because of prolonged asynchronous patterns of left ventricular contractions (3, 4, 5).

More than 80% of patients receiving PPMI are older than 65 years (2). Furthermore, patients with type 2 diabetes mellitus are associated with an increased risk of bradyarrhythmia. Rautio et al reported that the incidence of PPMI in diabetic patients was 242.2 /10,000 person-years compared to 152.5/10,000 person-years in the control subjects (6). Both advanced age (7) and diabetes mellitus (8) are associated with an increased risk of heart failure (HF) progression. Therefore, there is an urgent need to identify risk factors that can be used for accurate prediction of HF so that patients with greater risk of heart failure hospitalization (HFH) among the elderly diabetic patients receiving RVP can receive early interventions.

Malnutrition is associated with cardiac remodeling and unfavorable clinical outcomes in general population and in patients with heart failure (9, 10). Malnutrition and underweight are associated with a higher risk of adverse events in patients that have undergone PPMI (11, 12, 13, 14). The Prognostic Nutritional Index (PNI), the Geriatric Nutritional Risk Index (GNRI), the Naples Prognostic Score (NPS), and the Controlling Nutritional Status (CONUT) score are malnutrition screening tools that use easily available clinical information and biomarkers to estimate the nutritional status. However, the prognostic value of preimplantation nutritional status is not yet known for older diabetic patients receiving RVP.

A critical research hypothesis was generated that the preimplantation malnutrition status was associated with increased rate of HFH in older diabetic patients that received RVP. The study aimed to investigate the clinical value of the four malnutrition screening tools for the prediction of HFH in older diabetic patients that received RVP.

## Materials and Methods

### Ethic approval and study population

This retrospective observational cohort study was conducted between January 2017 and January 2018 at the Fuwai Hospital, Beijing, China. It was approved by the Ethics Committee of the Chinese Academy of Medical Sciences, Fuwai Hospital (Approval No. IRB2012-BG-006). The written informed consent was obtained from all the patients included in this study.

The inclusion criteria were as follows: (1) age ≥ 65 years; (2) diagnosed with diabetes mellitus; (3) underwent RVP for the first time. The diagnosis of diabetes mellitus was based on the previous history of hypoglycemic drugs or more than two records of fasting blood glucose levels ≥ 7.0 mmol/L during hospitalization. Initially, 1937 patients that received PPMI were enrolled during the study period. Then, we excluded patients with (1) age < 65 years (n = 742); (2) pacemaker upgrade or replacement (n = 297); (3) without diabetes mellitus (n = 665); and (4) values missing for total cholesterol (n = 2). Finally, 231 patients were included in our study (Figure 1).

**Figure 1:**
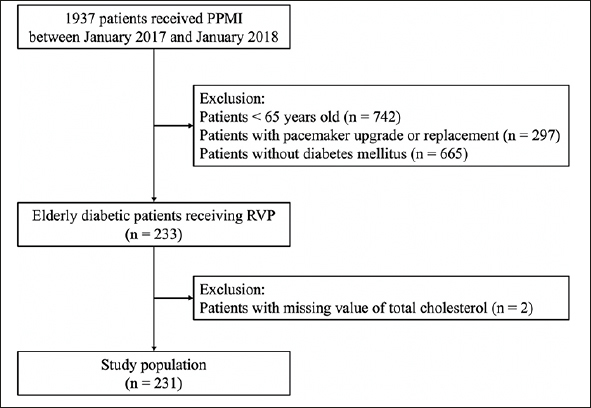
Flowchart for the selection of the study population

PPMI, permanent pacemaker implantation; RVP, right ventricular pacing.

### Assessment of nutritional status

The nutritional status of the included study subjects were assessed with four malnutrition screening indices using the clinical information and biomarkers acquired during admission.

PNI was calculated for all the patients using the following formula: 10 × albumin (g/dl) + 0.005 × total lymphocyte count (/mm3) (15). The patients were then categorized into the following groups: normal (PNI ≥ 45), mild (35 ≤ PNI < 45), and moderate-to-severe (PNI < 35) degree of malnutrition.

GNRI was calculated using the following formula: 1.489 × albumin (g/L) + 41.7 × current body weight/ideal body weight (16). The ideal body weight for males was defined as height (cm) − 100 − [height (cm) − 150]/4. The ideal body weight for females was defined as height (cm) − 100 − [height (cm) − 150]/2.5. Then, the patients were categorized into the following groups: normal (GNRI ≥ 98), mild (92 ≤ GNRI < 98), and moderate-to-severe (GNRI < 92) degree of malnutrition.

NPS scores were based on the following serum parameters: albumin, total cholesterol, neutrophil-to-lymphocyte ratio, and platelet-to-lymphocyte ratio (17). Patients were then categorized into the following groups: normal (NPR = 0), mild (NPR = 1–2), and moderate-to-severe (NPR = 3–4) degree of malnutrition. CONUT scores were estimated based on serum albumin, total cholesterol, and total lymphocyte counts (18). Patients were then categorized into the following groups: normal (CONUT = 0–1), mild (CONUT = 2–4), and moderate-to-severe (CONUT = 5–12) degree of malnutrition.

### Data collection

Trained study coordinators extracted the following clinical information for all the included patients from the electronic medical recording system: (1) demographic parameters such as age, gender, weight, height, body mass index (BMI), body fat proportion, smoke, and drink history; (2) clinical parameters such as heart rate (HR), systolic blood pressure (SBP), diastolic blood pressure (DBP), and New York Heart Association (NYHA) classification; (3) past medical history, including sinus node dysfunction (SND), atrioventricular block (AVB), atrial fibrillation (AF), and heart failure (HF); (4) medical therapy, including statin, angiotensin-converting enzyme inhibitor/ angiotensin receptor blocker (ACEi/ARB), β blocker, diuretic, insulin, a glucosidase, and metformin; (5) laboratory results for blood parameters, including the counts of white blood cells, neutrophils, lymphocytes, red blood cells, and platelets, as well as the serum levels of hemoglobin, albumin, total bilirubin, direct bilirubin, fasting blood glucose, estimated glomerular filtration rate (eGFR), blood urea nitrogen (BUN), total cholesterol (TC), high-density lipoprotein cholesterol (HDLC), low-density lipoprotein cholesterol (LDL-C), hemoglobin A1C (HbA1C), and N-terminal pro-brain natriuretic peptide (NT-pro BNP); (6) echocardiographic features such as left atrium diameter (LAD) and left ventricular ejection fraction (LVEF); and (7) details of RVS pacing, RVA pacing, and ventricular pacing burden.

### Follow-up and study endpoints

All the patients were followed up for any reports of HFH, which was defined as an unplanned outpatient or emergency department visit, or hospitalization because of new onset or worsening symptoms and signs of HF that required diuretic therapy and was accompanied with significantly elevated levels of N-terminal pro-B natriuretic peptide (NT-pro BNP). The duration of follow-up was measured from the date of receiving RVP to the date of the first HFH, or date of the follow-up deadline (January 30, 2022). The median follow-up duration was 53 months.

### Statistical analysis

Given an anticipated incidence rate of 20% for HF among older diabetic patients receiving right ventricular pacing (19), and targeting a statistical power of 80% at an alpha level of 0.05 in a two-sided hypothesis test, the study was designed to include 231 patients. The sample size calculation considers potential dropouts and non-response rates.

Continuous variables with normal distribution were represented as means ± standard deviation, and were compared using the independent sample t test. Continuous variables with skewed distribution were represented as median (25th quartile, 75th quartile), and were compared using the Wilcoxon rank sum test. Categorical variables were expressed as number (percentages) and were compared using the chi-squared test. Kaplan-Meier plots were used to determine the differences in the cumulative rates of HFH during the follow-up period in the normal, mild, and moderate-to-severe malnutrition groups. The survival data was compared using the log-rank test. Univariate Cox proportional hazard regression analysis was performed to identify the variables that showed significant correlation with HFH. These variables were then incorporated into the multivariate Cox regression models to determine the independent effects of malnutritional status on HFH based on the assessments from the four screening tools. The statistical data was analyzed using the R statistical software version 4.3.1. Two-tailed P value < 0.05 was considered statistically significant.

## Results

### Baseline clinical characteristics

This study included 231 older diabetic patients (51.5% males and 48.5% females) that had received RVP for the first time. The median age was 76 years. The median fasting blood glucose and HbA1C concentrations were 6.7 mmol/L (25th quartile = 5.6 mmol/L, 75th quartile = 8.3 mmol/L) and 6.9% (6.3%, 7.7%), respectively. The pacing leads were placed within the RVS and the RVA in 35.1% and 64.9% of the study subjects, respectively. The ventricular pacing percentage ≥ 40% was observed in 71.4% of the study subjects. In the study cohort, 6.1% of patients were classified as NYHA II-IV before implantation. Furthermore, the median NT-pro BNP concentration was 357.7 pg/ml (158.6 pg/ml, 954.6 pg/ml) pg/ml, and the median LVEF was 63.0% (60.0%, 65.0%) before implantation. During the median follow-up period of 53 months, 19.9% of the patients revisited the hospital due to HF (Table 1).

**Table 1:** Baseline characteristics and preimplantation malnutrition of the study population

**Variables**	**Total (n = 231)**
Age, years	76.0 (71.0, 79.0)
Male, n (%)	119 (51.5)
Weight, kg	68.0±12.2
Height, cm	165.0 (158.0, 170.0)
BMI, kg/m^2^	24.9 (22.6, 27.3)
BMI classification, n (%)	
Underweight	9 (3.9)
Normal	70 (30.3)
Overweight	110 (47.6)
Obesity	42 (18.2)
Body fat proportion, %	35.7 (32.1, 41.4)
Smoke, n (%)	77 (33.3)
Drink, n (%)	64 (27.7)
HR, /min	65.0 (60.0, 77.5)
SBP, mmHg	140.0 (128.5, 154.0)
DBP, mmHg	70.0 (60.0, 75.0)
NYHA II-IV, n (%)	14 (6.1)
Past medical history, n (%)	
SND	154 (66.7)
AVB	78 (33.8)
AF with slow HR	20 (8.7)
Hypertension	199 (86.1)
Coronary artery disease	128 (55.4)
HF	50 (21.6)
Medical therapy, n (%)	
Statin	174 (75.3)
ACEi/ARB	138 (59.7)
? blocker	129 (55.8)
Diuretic	79 (34.2)
Insulin	65 (28.1)
a glucosidase	141 (61)
Metformin	65 (28.1)
Laboratory results	
White blood cell, × 10^9^/L	6.5 (5.5, 8.0)
Neutrophil, × 10^9^/L	4.0 (3.2, 5.1)
Lymphocyte, × 10^9^/L	1.7 (1.3, 2.2)
Red blood cell, × 10^9^/L	4.3 (3.9, 4.8)
Hemoglobin, g/L	130.4±18.4
Platelet, × 10^9^/L	197 (154, 228)
Albumin, g/L	41.8 (38.6, 45.6)
Total bilirubin, µmol/L	13.7 (10.7,18.1)
Direct bilirubin, µmol/L	2.6 (1.9, 3.3)
Fasting blood glucose, mmol/L	6.7 (5.6, 8.3)
eGFR, ml/min/1.73m^2^	68.9 (58.7, 77.0)
BUN, mmol/L	6.5 (5.1, 8.4)
TC, mmol/L	3.8 (3.3, 4.4)
HDL-C, mmol/L	1.1 (0.9, 1.4)
LDL-C, mmol/L	2.1 (1.7, 2.6)
HbA1C, %	6.9 (6.3, 7.7)
NT-pro BNP, pg/mL	357.7 (158.6, 954.6)
Echocardiography features	
LAD, mm	40.0 (36.0, 43.0)
LVEF, %	63.0 (60.0, 65.0)
Pacing details, n (%)	
Right ventricular septum	81 (35.1)
Right ventricular apex	150 (64.9)
Ventricular pacing ≥ 40%	165 (71.4)
Malnutrition screening tools	
*PNI groups*	
Normal	189 (81.8)
Mild	39 (16.9)
Moderate-to-severe	3 (1.3)
*GNRI groups*	
Normal	196 (84.8)
Mild	25 (10.8)
Moderate-to-severe	10 (4.3)
*NPS groups*	
Normal	31 (13.4)
Mild	177 (76.6)
Moderate-to-severe	23 (10.0)
*CONUT groups*	
Normal	78 (33.8)
Mild	135 (58.4)
Moderate-to-severe	18 (7.8)
HF hospitalization, n (%)	46 (19.9)


Abbreviations: BMI, body mass index; HR, heart rate; SBP, systolic blood pressure; DBP, diastolic blood pressure; SND, sinus node dysfunction; AVB, atrioventricular block; NYHA, New York Heart Association; AF, atrial fibrillation; HF, heart failure; ACEi/ARB, angiotensin-converting enzyme inhibitor/angiotensin receptor blocker; eGFR, estimated glomerular filtration rate; BUN, blood urea nitrogen; TC, total cholesterol; HDL-C, high-density lipoprotein cholesterol; LDL-C, low-density lipoprotein cholesterol; HbA1C, hemoglobin A1C; NT-pro BNP, N-terminal pro-brain natriuretic peptide; LAD, left atrium diameter; LVEF, left ventricular ejection fraction; PNI, the Prognostic Nutritional Index; GNRI, the Geriatric Nutritional Risk Index; NPS, the Naples Prognostic Score; CONUT, the Controlling Nutritional Status.

### Clinical features of preimplantation malnutrition

As shown in Table 1, 18.2%, 15.2%, 86.6%, and 66.2% of the patients were diagnosed with malnutrition before implantation based on the PNI, GNRI, NPS, and CONUT score, respectively.

Patients with preimplantation malnutrition were associated with older age, higher proportion of males, β blocker usage, preexisting coronary artery disease, HF or AF with slow HR, lower proportion of metformin use and SND, higher values for height, neutrophils, BUN, direct bilirubin, NT-pro BNP, and LAD, lower values for BMI, proportion of body fat, lymphocytes, red blood cells, hemoglobin, platelets, albumin, TC, HDL-C, LDL-C, total bilirubin, and HbA1C (Table 2).

**Table 2:** The clinical characteristics of malnutrition

		**PNI**			**GNRI**			**NPS**			**CONUT**	
**Variable**	**Normal**	**Malnutrition**	**P**	**Normal**	**Malnutrition**	**P**	**Normal**	**Malnutrition**	**P**	**Normal**	**Malnutrition**	**P**
Age, years	75.0 (70.0,78.0)	80.0 (75.0, 85.0)	<0.001	75.0 (70.0,78.0)	80.0 (75.0, 85.0)	<0.001	74.0 (70.0,77.5)	76.0 (71.0,79.3)	0.193	74.0 (70.0,78.8)	76.0 (72.0, 80.0)	0.033
Male, n (%)	94 (49.7)	25 (59.5)	0.328	98 (50)	21 (60)	0.365	10 (32.3)	109 (54.5)	0.035	34 (43.6)	85 (55.6)	0.114
Weight, kg	68.0 + 12.8	68.0+9.1	0.976	69.0 ± 12.2	62.6 ± 11.2	0.003	65.3 ± 12.4	68.4 ± 12.2	0.201	68.0 ± 12.0	69.0 ± 12.4	0.990
Height, cm	164.0 (158.0, 170.0)	168.0 (160.0, 173.8)	0.029	164.0 (158.0, 170.0)	168.0 (159.5, 175.5)	0.026	162.0 (156.0, 168.5)	165.0 (158.0, 170.3)	0.082	163.0 (158.0, 168.0)	165.0 (158.0, 172.0)	0.084
BMI, kg/m2	25.1 (22.9, 27.4)	24.3 (22.4, 26.0)	0.121	25.7 (23.7, 27.7)	22.3 (21.1, 24.2)	<0.001	24.7 (23.0,27.1)	25.0 (22.6, 27.3)	0.748	25.4 (23.5, 27.9)	24.8 (22.3,27.1)	0.125
Body fat proportion, %	35.9 (32.3, 41.8)	35.2 (30.6, 40.3)	0.380	36.4 (32.7,42.0)	33.0 (28.4, 37.6)	<0.001	39.2 (33.4,42.0)	35.5 (31.9,41.1)	0.177	37.8 (32.9,41.7)	35.1 (31.4,40.6)	0.054
Past medical history, n (%)												
SND	129 (68.3)	25 (59.5)	0.366	134 (68.4)	20 (57.1)	0.270	24 (77.4)	130 (65.0)	0.246	60 (76.9)	94 (61.4)	0.027
AF with slow HR	16 (8.5)	4 (9.5)	1.000	17 (8.7)	3 (8.6)	1.000	2 (6.5)	18 (9.0)	0.900	2 (2.6)	18 (11.8)	0.035
Coronary artery disease	101 (53.4)	27 (64.3)	0.268	107 (54.6)	21 (60.0)	0.683	11 (35.5)	117 (58.5)	0.027	37 (47.4)	91 (59.5)	0.109
HF	32 (16.9)	18 (42.9)	<0.001	39 (19.9)	11 (31.4)	0.193	5 (16.1)	45 (22.5)	0.571	12 (15.4)	38 (24.8)	0.139
Medical therapy n (%)												
β blocker	98 (51.9)	31 (73.8)	0.016	104 (53.1)	25 (71.4)	0.067	17 (54.8)	112 (56.0)	1.000	41 (52.6)	88 (57.5)	0.564
Metformin	59 (31.2)	6 (14.3)	0.044	59 (30.1)	6 (17.1)	0.172	9 (29.0)	56 (28.0)	1.000	23 (29.5)	42 (27.5)	0.864
Laboratory results												
Neutrophil, × 107L	3.8 (3.1,4.9)	4.8 (3.6, 6.4)	0.004	3.9 (3.1,5.0)	4.7 (3.7, 5.5)	0.020	3.5 (2.9, 4.8)	4.0 (3.2, 5.1)	0.117	3.8 (3.1,4.9)	4.0 (3.2, 5.2)	0.157
Lymphocyte, × 10^9^/L	1.8 (1.4, 2.2)	1.2 (0.9, 1.6)	<0.001	1.8 (1.4, 2.2)	1.4 (1.1, 2.0)	0.024	1.9 (1.7, 2.3)	1.6 (1.3, 2.1)	0.003	2.1 (1.8, 2.3)	1.5 (1.2, 1.9)	<0.001
Red blood cell, × 10^S^/L	4.4 (4.0,4.8)	3.8 (3.4, 4.2)	<0.001	4.4 (3.9,4.8)	4.0 (3.4, 4.2)	<0.001	4.4 (4.0,4.6)	4.3 (3.8, 4.8)	0.616	4.4 (4.0, 4.8)	4.2 (3.6, 4.7)	0.057
Hemoglobin, g/L	132.7 ± 17.3	120.0 ± 19.8	<0.001	132.3 ± 18.1	119.4 ± 16.0	<0.001	130.6 ± 14.1	130.3 ± 19.0	0.933	134.0 ± 18.1	128.5 ± 18.3	0.031
Platelet, × 10^p^/L	199.0 (155.0, 234.0)	184.0 (148.5, 217.0)	0.453	198.0 (154.8, 229.3)	182.0 (145.0, 224.0)	0.635	210.0 (191.5, 227.0)	191.5 (149.5, 229.3)	0.132	210.0 (182.8, 256.0)	185.0 (148.0, 218.0)	<0.001
Albumin, g/L	43.1 (40.2, 46.1)	34.9 (32.9,36.8)	<0.001	42.9 (39.8,46.0)	35.0 (32.9, 36.7)	<0.001	44.0 (40.4,45.1)	41.6 (38.0,45.6)	0.129	44.1 (40.0, 46.4)	41.2 (36.9,45.0)	<0.001
Total bilirubin, µmol/L	14.0 (11.2, 18.4)	12.1 (8.9, 17.8)	0.058	14.1 (11.3, 18.4)	10.8 (8.7, 15.2)	0.003	13.2 (10.5, 15.8)	13.9 (10.8, 18.6)	0.225	13.6 (10.7, 17.1)	13.9 (10.8, 18.8)	0.438
Direct bilirubin, //mol/L	2.6 (1.9,3.3)	2.6 (1.9,3.7)	0.870	2.6 (2.0,3.3)	2.1 (1.8,3.4)	0.230	2.1 (1.7, 2.7)	2.6 (2.0, 3.5)	0.011	2.3 (1.7, 3.1)	2.6 (2.0, 3.6)	0.004
BUN, mmol/L	6.3 (5.1, 8.0)	7.9 (6.0, 10.1)	0.002	6.5 (5.1,8.3)	6.8 (5.4, 9.5)	0.300	6.6 (5.2, 7.9)	6.5 (5.1, 8.4)	0.993	6.7 (5.4, 8.0)	6.4 (5.1, 8.6)	0.653
TC, mmol/L	3.8 (3.3, 4.4)	3.5 (3.0,3.8)	0.003	3.8 (3.3, 4.4)	3.7 (3.1,3.9)	0.099	5.2 (5.0, 5.7)	3.6 (3.2,4.1)	<0.001	4.4 (4.0, 5.1)	3.4 (2.9,3.8)	<0.001
HDL-C, mmol/L	1.1 (0.9, 1.4)	1.1 (0.8, 1.4)	0.271	1.1 (0.9, 1.4)	1.1 (0.9, 1.4)	0.707	1.3 (1.1, 1.8)	1.1 (0.9, 1.3)	0.002	1.2 (1.0, 1.5)	1.1 (0.9, 1.3)	<0.001
LDL-C, mmol/L	2.2 (1.7, 2.7)	2.0 (1.6, 2.3)	0.033	2.1 (1.7, 2.7)	2.1 (1.7, 2.5)	0.529	3.4 (3.1,3.8)	2.0 (1.6, 2.5)	<0.001	2.8 (2.3, 3.4)	1.9 (1.5, 2.2)	<0.001
HbAlC, %	6.9 (6.3, 7.8)	6.7 (6.2, 7.4)	0.171	70 (6.4,79)	6.5 (6.0, 7.2)	0.008	7.0 (6.3, 7.7)	6.9 (6.3, 7.7)	0.990	6.8 (6.3, 7.4)	6.9 (6.3, 7.8)	0.216
NT-pro BNP, pg/mL	270.1 (135.2, 667.0)	10470 (612.4, 1279.0)	<0.001	278.5 (134.4, 826.0)	917.3 (438.1, 1181.0)	<0.001	175.8 (102.1, 332.6)	398.1 (186.4, 985.5)	0.008	204.6 (106.0,449.0)	486.8 (198.3, 1090.0)	<0.001
Echocardiography features												
LAD, mm	40.0 (36.0,43.0)	41.5 (39.0,44.8)	0.024	40.0 (36.0, 43.0)	39.0 (36.5,43.0)	0.621	38.0 (35.0,41.0)	40.0 (37.0, 43.3)	0.080	38.0 (35.0, 41.0)	41.0 (38.0,44.0)	<0.001


Abbreviations: PNI, the Prognostic Nutritional Index; GNRI, the Geriatric Nutritional Risk Index; NPS, the Naples Prognostic Score; CONUT, the Controlling Nutritional Status; BMI, body mass index; SND, sinus node dysfunction; AF, atrial fibrillation; HR, heart rate; HF, heart failure; BUN, blood urea nitrogen; TC, total cholesterol; HDL-C, high-density lipoprotein cholesterol; LDL-C, low-density lipoprotein cholesterol; HbAlC, hemoglobin A1C; NT-pro BNP, N-terminal pro-brain natriuretic peptide; LAD, left atrium diameter.

### Preimplantation malnutrition is associated with heart failure hospitalization

The cumulative rate of HFH during the follow-up period was significantly higher in patients with preimplantation malnutrition as identified by PNI (log-rank = 13.0, P = 0.001), GNRI (log-rank = 8.5, P = 0.01), and NPS (log-rank = 15.7, P < 0.001) compared to those with normal nutritional status, but was statistically insignificant based on the CONUT score (logrank = 2.7, P = 0.3) (Figure 2).

**Figure 1:**
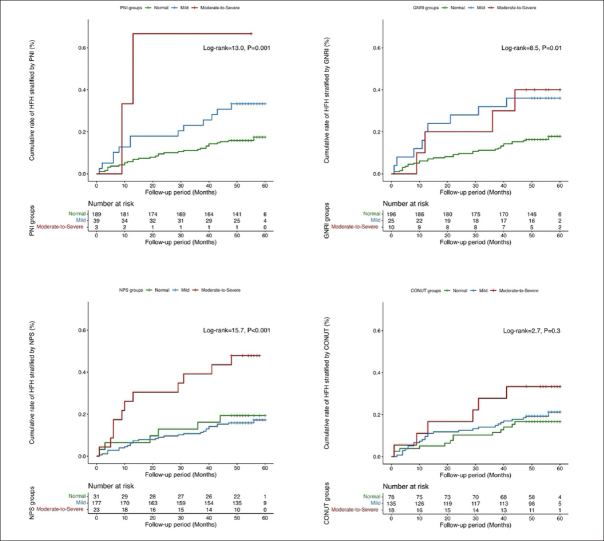
The cumulative rate of HF hospitalization stratified by nutritional screening tools

HF, heart failure; PNI, the Prognostic Nutritional Index; GNRI, the Geriatric Nutritional Risk Index; NPS, the Naples Prognostic Score; CONUT, the Controlling Nutritional Status.

Univariate Cox proportional hazard regression analysis was performed to assess the prognostic significance of clinical indicators on HFH based on the hazard ratios. Univariate analysis results showed that age (HR = 1.06, 95% CI: 1.01–1.11, P = 0.019), NYHA II-IV (HR = 3.54, 95% CI: 1.59–7.92, P = 0.002), preexisting HF (HR = 4.66, 95% CI: 2.61–8.33, P < 0.001), use of β blocker (HR = 2.17, 95% CI: 1.14–4.12, P = 0.018), lg (NT-pro BNP) level (HR = 2.02, 95% CI: 1.53–2.67, P < 0.001), LAD (HR = 1.11, 95% CI: 1.06–1.16, P < 0.001), and LVEF (HR = 0.91, 95% CI: 0.87–0.95, P < 0.001) were associated with HFH (Table 3).

**Table 3:** Unadjusted and adjusted HR for HF hospitalization

**Variables**	**Unadjusted**			Adjusted*		
	**HR**	**95% CI**	**P**	**HR**	**95% CI**	**P**
Age	1.06	1.01–1.11	0.019			
NYHA II-IV	3.54	1.58–7.92	0.002			
Preexisting HF	4.66	2.61–8.33	<0.001			
β blocker	2.17	1.14–4.12	0.018			
lg (NT-pro BNP)	2.02	1.53–2.67	<0.001			
LAD	1.11	1.06–1.16	<0.001			
LVEF	0.91	0.87–0.95	<0.001			
PNI	0.94	0.91–0.98	0.002	0.97	0.93–1.01	0.142
GNRI	0.96	0.93–0.99	0.004	0.97	0.936–0.997#	0.032
NPS	1.53	1.13–2.05	0.005	1.11	0.80–1.55	0.538
CONUT	1.20	1.01–1.42	0.036	0.97	0.80–1.19	0.778
PNI groups						
Normal	/			/		
Mild	2.23	1.16–4.26	0.016	0.89	0.42–1.89	0.763
Moderate-to-severe	6.69	1.59–28.15	0.010	4.66	1.03–21.00	0.045
GNRI groups						
Normal	/			/		
Mild	2.48	1.18–5.18	0.016	1.81	0.84–3.90	0.128
Moderate-to-severe	2.62	0.93–7.39	0.069	3.02	1.02–9.00	0.047
NPS groups						
Normal	/			/		
Mild	0.82	0.34–1.97	0.650	0.59	0.24–1.45	0.252
Moderate-to-severe	3.03	1.11–8.20	0.029	1.03	0.35–3.03	0.962
CONUT groups						
Normal	/			/		
Mild	1.23	0.63–2.38	0.542	0.82	0.41–1.65	0.578
Moderate-to-severe	2.22	0.84–5.83	0.107	0.74	0.26–2.10	0.569


*Pre-existing HF, use of β blocker, lg NT-pro BNP level, LAD, and LVEF were adjusted in the multivariate model. #CI was presented with greater precision due to its proximity to 1.00. Abbreviations: HR, hazard ratio; CI, confidential interval; HF, heart failure; HR, hazard ratio; CI, confidential interval; NYHA, New York Heart Association; lg (NT-pro BNP), the common logarithm of N-terminal pro-brain natriuretic peptide; LAD, left atrium diameter; LVEF, left ventricular ejection fraction; PNI, the Prognostic Nutritional Index; GNRI, the Geriatric Nutritional Risk Index; NPS, the Naples Prognostic Score; CONUT, the Controlling Nutritional Status.

Subsequently, multivariate Cox proportional regression models were constructed by integrating the significant risk factors for HFH based on the results from the univariate regression analysis and all the nutritional indices. As continuous variables, all the nutritional indices showed significant correlation with HFH (all P < 0.05). However, only GNRI was independently associated with HFH (HR = 0.97, 95% CI: 0.937–0.997, P = 0.032). As categorical variables, PNI, GNRI, and NPS showed significant correlation with HFH (P < 0.05). After adjustment of potential confounding factors, including preexisting HF, use of β blocker, lg (NT-pro BNP) level, LAD, and LVEF, moderate-to-severe degree of malnutrition based on the PNI (HR = 4.66, 95% CI: 1.03–21.00, P = 0.045), and GNRI (HR = 3.02, 95% CI: 1.02–9.00, P = 0.047) were independently associated with HFH (Table 3).

## Discussion

This study showed that preimplantation malnutrition was common in older diabetic patients receiving RVP. The cumulative rate of HFH was significantly higher for patients with preimplantation malnutrition according to the PNI, GNRI, and NPS data. As a continuous variable, only GNRI was independently associated with HFH. As categorical variables, after adjusting for the potential confounding covariates, the moderate-to-severe degree of malnutrition assessment based on PNI and GNRI data was independently related with HFH. However, malnutrition assessments based on the NPS and CONUT score were not associated with increased risk for HFH.

RVP is the main pacing strategy for patients with high-degree AVB and SND (2, 20, 21). In several cases, prolonged ventricular pacing caused asynchrony of the left ventricular contraction and gradually reduced cardiac function (22). Advanced age (7) and diabetes mellitus (8) were significant risk factors associated with HF progression. Therefore, early identification of the high-risk population is critical for improving the prognosis in older diabetic patients with RVP.

Malnutrition is associated with adverse clinical outcomes in cardiovascular diseases but is often neglected as a risk factor (9, 10). Echouffo-Tcheugui et al (14) and Yanagisawa et al (23) reported that underweight patients with HF that received implantable cardioverter-defibrillator (ICD) therapy were associated with poorer prognosis. Hsu et al analyzed global population cohort that received ICD and reported significantly higher rate of adverse events in the underweight recipients (13). Balli et al (12) and Yamaguchi et al (11) analyzed patients with bradycardia that received PPMI and demonstrated a positive correlation between the preprocedural malnutritional status and postoperative development of pacing-induced cardiomyopathy and all-cause mortality. However, very few studies have investigated the prognostic value of multiple malnutrition screening tools in older diabetic patients with RVP. In our study population, malnutrition was a common comorbidity in older diabetic patients that received PPMI. Furthermore, the cumulative rate of HFH was significantly higher in the malnutrition group.

Previous studies have reported the relationship between malnutrition and HF development (24, 25). HF is characterized by progressive cardiac remodeling, with inflammation and fibrosis playing a key role (26, 27). Dietary supplementation with n-3 polyunsaturated fatty acids induces anti-inflammatory and anti-oxidative effects that suppress the onset of HF (28). Vitamin D alleviates cardiac hypertrophy, fibrosis, and dysfunction (29, 30). ATP depletion and excessive generation of reactive oxygen species because of thiamine deficiency impaired myocardial contractility and resulted in HF (31, 32).

The risk of malnutrition varies between different settings. Moreover, the prevalence of malnutrition varies based on the type of malnutrition screening tools used. Therefore, it is recommended that an individual nutritional screening approach that is suitable for a specific population should be used to estimate malnutrition with consistency and accuracy (33). Previous studies have demonstrated the prognostic values of various malnutrition screening tools for the prediction of adverse outcomes in patients with PPMI.

GNRI was initially used to evaluate the risk of morbidity and mortality associated with malnutrition in the hospitalized geriatric patients (16, 34). The severe malnutritional status based on the GNRI analysis was an independent predictor of all-cause mortality in patients with bradycardia after receiving PPMI (HR = 4.49, P < 0.001) (11). Our results also showed that moderate-to-severe degree of malnutrition based on GNRI was independently associated with HFH. As a continuous variable, GNRI showed significant negative correlation with HFH.

PNI was originally used for evaluating older patients with cancer that underwent elective surgery (15). Balli et al demonstrated the negative relationship between PNI and pacing-induced cardiomyopathy in patients that underwent PPMI (12). The decreased PNI values relative to the baseline PNI values were independently associated with unfavorable outcomes (35). Likewise, our study demonstrated that moderate-to-severe malnutrition was an independent predictor of HFH.

On the contrary, the NPS value and CONUT score were not associated with HFH. This may be caused by the variables that were used by these tools to estimate the nutritional status. Both NPS and CONUT score use serum total cholesterol as one of the parameters to estimate malnutrition, and total cholesterol levels above 180 mg/dL (4.65 mmol/L) were regarded as normal. However, high cholesterol levels are significantly associated with increased risk of HF (36, 37, 38). Furthermore, cumulative prior exposure to LDL-C was significantly associated with an increased risk of incident cardiovascular events, including HF (39). Therefore, HF patients that were considered to have a normal nutritional status based on the NPS and CONUT score, may be at a higher risk of HFH.

Our study has several limitations Firstly, this was a retrospective study. Therefore, the results of this study should be interpreted with caution and would require further analysis in randomized-controlled prospective studies. Secondly, the malnutrition screening tools included in our study incorporated simple acquirable biomarkers from the clinical settings. In the future, information regarding weight loss, mobility, and neuropsychological problems should be assessed for a thorough and detailed evaluation of the nutritional status (40). Finally, although covariates were adjusted to reduce confounding effects in the multivariate regression analyses, there might be residual confounding factors.

## Conclusions

Preimplantation malnutrition was highly prevalent in older diabetic patients receiving RVP and was associated with an increased risk of HFH. The prevalence and prognostic value of malnutrition showed significant differences among different malnutrition screening tools compared in this study. Our results showed that the PNI and GNRI could be feasible for rapid evaluation and risk stratification of the patients, based on nutritional status. These malnutrition screening tools showed high prognostic value for accurately predicting HFH in older diabetic patients with RVP.

## Data Availability

The raw data supporting the conclusions of this article will be made available by the authors, without undue reservation.
